# A Comparative Perspective on Three Primate Species’ Responses to a Pictorial Emotional Stroop Task

**DOI:** 10.3390/ani11030588

**Published:** 2021-02-24

**Authors:** Lydia M. Hopper, Matthias Allritz, Crystal L. Egelkamp, Sarah M. Huskisson, Sarah L. Jacobson, Jesse G. Leinwand, Stephen R. Ross

**Affiliations:** 1 Lester E. Fisher Center for the Study and Conservation of Apes, Lincoln Park Zoo, Chicago, IL 60614, USA; crystallegelkamp@gmail.com (C.L.E.); shuskisson@lpzoo.org (S.M.H.); sjacobson1112@gmail.com (S.L.J.); jleinwand@lpzoo.org (J.G.L.); sross@lpzoo.org (S.R.R.); 2 School of Psychology and Neuroscience, University of St Andrews, St Andrews KY16 9AJ, UK; ma249@st-andrews.ac.uk; 3 Psychology, Graduate School and University Center, City University of New York, New York, NY 10016, USA

**Keywords:** affect, attentional bias, cognitive bias, emotions, snake detection theory, stroop effect, touchscreen, welfare, zoo

## Abstract

**Simple Summary:**

As animals cannot tell us how they feel, we must develop tests to make inferences about how they are feeling to assess their welfare. Here, we adapted a task that has been previously used with humans and chimpanzees to assess how chimpanzees, gorillas, and Japanese macaques respond to pictures of different emotional valences. Specifically, if the primates perceive emotionally arousing photographs differently as compared to neutral photographs, we would expect them to “trip up” more when responding to emotional stimuli. We presented the primates with a touchscreen task in which they had to select one of two differently colored squares. However, the squares always contained either positive photographs (a preferred food), negative photographs (a snake), or neutral photographs (human-made objects). The primates made more errors in selecting the correct square when the squares contained positive or negative photographs as compared to neutral photographs, indicating that they perceived these differentially, and that this caused them to make mistakes. Thus, the primates’ cognitive ability was disrupted by emotional stimuli. This offers important insights into how primates perceive the world around them, and how they process elements that may have negative or positive associations.

**Abstract:**

The Stroop effect describes interference in cognitive processing due to competing cognitive demands. Presenting emotionally laden stimuli creates similar Stroop-like effects that result from participants’ attention being drawn to distractor stimuli. Here, we adapted the methods of a pictorial Stroop study for use with chimpanzees (N = 6), gorillas (N = 7), and Japanese macaques (N = 6). We tested all subjects via touchscreens following the same protocol. Ten of the 19 subjects passed pre-test training. Subjects who reached criterion were then tested on a standard color-interference Stroop test, which revealed differential accuracy in the primates’ responses across conditions. Next, to test for an emotional Stroop effect, we presented subjects with photographs that were either positively valenced (a preferred food) or negatively valenced (snakes). In the emotional Stroop task, as predicted, the primates were less accurate in trials which presented emotionally laden stimuli as compared to control trials, but there were differences in the apes’ and monkeys’ response patterns. Furthermore, for both Stroop tests, while we found that subjects’ accuracy rates were reduced by test stimuli, in contrast to previous research, we found no difference across trial types in the subjects’ response latencies across conditions.

## 1. Introduction

The Five Freedoms model of animal welfare [1] was developed from the tenet that animal welfare is defined as an animal’s ability to respond to, and cope with, their environment [2]. However, the Five Freedoms model is limited in that four of the five requirements only consider animals’ need to be *free from* negative or harmful experiences (e.g., freedom from thirst, hunger, and malnutrition; freedom from fear and distress), while the fifth requirement is the freedom to express normal behavior. In this way, the Five Freedoms model prioritizes the absence of negative experiences rather than a promotion of positive well-being. Moreover, even under ideal or ‘natural’ conditions, an animal is unlikely to be free from all negative experiences at all times. Thus, despite being widely adopted, the Five Freedoms model nonetheless has limitations [3].

Accordingly, in the last 10–15 years, there has been a move towards understanding animal welfare in a more holistic manner, specifically one that seeks to create conditions in which animals *thrive* physically and emotionally, not just *cope* [4]. Following this, animal welfare researchers have sought to identify indicators of *positive animal welfare* (e.g., [5,6]). A key foundation of the positive animal welfare movement is the principle that animals have emotional repertoires similar to those of humans and that animals can experience both negative emotions (e.g., fear and panic) as well as positive emotions (e.g., euphoria and comfort). Thus, we can promote conditions in which animals are more likely to experience positive, rather than negative, emotions to enhance their welfare [7]. People aim to achieve this via the provision of safe living conditions, complex and enriching habitats, and species-typical social groups, although evaluating the efficacy of such interventions is often a complex task.

As nonverbal animals cannot report on their own internal affective state, animal welfare scientists seek outputs that they can measure to make inferences about an animal’s welfare. However, measurable outputs, and in particular behavioral measures, do not always reliably map onto an animal’s internal state due to equifinality (the same output can arise as a result of different inputs or experiences) and multifinality (the same inputs can create different outputs). Indeed, it has been noted that the field of positive animal welfare currently lacks reliable definitions and objective measures [8], so we should seek to develop new assessment tools to measure positive animal welfare and responses to emotionally valent stimuli [9]. One approach would be to seek a deeper understanding as to whether and how animals’ emotional states influence their cognitive processing [10,11]. An increasingly popular method to address this is via the application of cognitive bias tests. Attention bias and judgment bias tests have been adapted for use with a range of nonhuman animal species, including honeybees [12], rats [13], sheep [14], and nonhuman primates [15]. In general terms, such tests aim to assess whether an animal’s mood interferes with their ability to attend to information or the expectations they have about task outcomes. Furthermore, these protocols offer ways to assess changes in animals’ cognitive processing across different conditions or states [15,16,17] or in relation to other measures of well-being [18]. It has been pointed out, however, that for tasks to be successful in the long term, their reliability with regard to measuring individual differences still needs to be established [19].

In addition to studying how animals’ responses to stimuli are mediated by mood, another way to explore animals’ emotional experiences is to test their response to emotionally valenced stimuli [9]—specifically, to evaluate whether animals respond differentially to emotional, as compared to neutral, stimuli, or whether they show different reactions to positively and negatively valenced stimuli (e.g., [20]). One method that has been commonly used with humans to address such questions is the emotional Stroop task, which was developed as an adaptation of early tests of the Stroop effect. The Stroop effect describes the *interference* in an individual’s attention due to the presentation of incongruent stimuli. A classic example of such interference is seen when human participants show a reduction in reading fluency when asked to read the names of colors printed in a different color ink (e.g., reading the word “green” written in yellow ink, or the word “blue” written in red ink) [21]. This is because the participant’s ability to perform the target task (read the words) is being interfered with by a second, distractor task (parse the colors of the words). In contrast, the emotional Stroop task consists of emotion-based words (e.g., crash, grief, or death) intermixed with neutral words (e.g., gate, clock, or heard) (e.g., [22]), sometimes presented in combination with images (e.g., [23]), that participants must read. The emotional Stroop effect creates a failure in selective *attention* via the presentation of emotionally arousing stimuli [24,25]. Reflecting an evolutionarily adaptive response, a participant’s attention is drawn to emotionally relevant stimuli, causing them to prioritize processing them, which can lead to reduced task accuracy (reviewed in [25]) or delayed response times due to a “cognitive freeze” effect [26]. Thus, it is likely that the mechanisms underlying impaired performance in classic Stroop paradigms (cognitive interference) differ from that in emotional Stroop tasks (selective attention or “emotional intrusion”) [22].

Although commonly explored in humans, only a few tests of the Stroop effect have been administered with nonhuman animals, with the majority testing primates (e.g., [27,28,29,30]). Moreover, to our knowledge, only one study has adapted the emotional Stroop task for use with nonhuman animals [27]. Allritz et al. [27] tested chimpanzees (*Pan troglodytes*) with a pictorial version of the emotional Stroop task that presented different photographs, each aimed to elicit different affective responses. This approach builds off methods used with human participants in which images of different valences have been shown to interfere with participants’ response accuracy or latencies [31,32]. The study by Allritz and colleagues [27] first revealed that, in a standard Stroop test, chimpanzees were less accurate and had increased response latencies in incongruent trials, compared to congruent or control trials, suggestive of a Stroop-like effect in their cognitive processing. Second, in the emotional Stroop test, the chimpanzees had increased response latencies when selecting negatively valenced stimuli as compared to controls, although accuracy was not impacted by stimuli type. Allritz et al. [27] concluded that their pictorial Stroop test represented a reliable way to test the relationship between emotion and cognition, and one that can easily be implemented with primates, but that the paradigm may not be the best method to study the underlying mechanism of such interactions.

The aim of our study was to adapt the methods used by Allritz et al. [27] for use with another population of chimpanzees and to test species that have not been tested previously on their response to an emotional Stroop task (*Gorilla gorilla gorilla* and *Macaca fuscata*). By using the same methods across three species, we sought to determine whether Stroop-like effects may be universal across primate species and to gain further information about the potential influence of differently valenced stimuli on their cognitive processing. To do so, we first tested all our subjects with a standard Stroop task using differently colored, neutrally valenced images (geometric shapes). We predicted that, as has been shown previously for macaques and chimpanzees, the primates would show reduced accuracy and increased response latencies when presented with incongruent as compared to congruent stimuli due to cognitive interference. Next, to determine whether selective attention would arise from presenting differently valenced stimuli, we predicted that the subjects would show reduced accuracy and increased response times in trials that showed emotionally valenced stimuli, as compared to neutral stimuli.

## 2. Methods

### 2.1. Subjects and Housing

We tested 19 zoo-housed primates: six chimpanzees (4 females, 2 males, average age: 25.7 years, s.d. = 5.76), seven gorillas (2 females, 5 males, average age: 15.44 years, s.d. = 7.72), and six Japanese macaques (5 females, 1 male, average age: 9.17 years, s.d. = 5.56) (Table 1). All 19 primates lived in social groups at Lincoln Park Zoo, Chicago, USA. One of the female chimpanzees lived in a social group with two additional females and two males, who did not participate in this study, while the remaining five chimpanzees lived together in a second social group with one additional nonparticipating female. Four of the male gorillas lived together in an all-male group while the remaining three gorillas lived in a mixed-sex family group comprised of one male and six females (including the subjects). Finally, the six macaque subjects lived with an additional five monkeys (three females, two males) in a group of 11. All the primates’ habitats were comprised of indoor and outdoor areas (including off-exhibit spaces) that provided multiple climbing structures, elevated sleeping areas, and visual barriers. The primates were all fed daily with both fresh produce and primate chow and were also given enrichment devices each day (for further details about their housing and husbandry, see [16,33]).

### 2.2. General Testing Protocol

Across all test phases, described in detail below, our general approach to testing the subjects remained constant. We tested all subjects using 10-point 55 cm capacitive ViewSonic LCD touchscreen monitors (1920 × 1080 resolution) using Zenrichment ApeTouch software Version 11.0. We tested all subjects in their home habitat, either in their main exhibit in public view or in an adjacent off-exhibit holding area, where they could stay within visual, olfactory, and auditory range of the rest of their group [34]. We tested the apes on a touchscreen monitor that was mounted on a mobile and adjustable cart that allowed us to set the screen at eye level for each subject depending on their size [16]. We began each subject’s test session by placing the touchscreen flush against their enclosure mesh and verbally inviting them to participate. The subject initiated their session when they touched the screen. We terminated a session when a subject did not approach the touchscreen within five minutes, stopped participating for five minutes, or finished the maximum-allowed number of trials for that day, which differed across test phases (see below for details). If the subject was interrupted by another group member, we paused the session, and gave them another chance to participate once the “interrupter” moved away.

**Figure 1:**
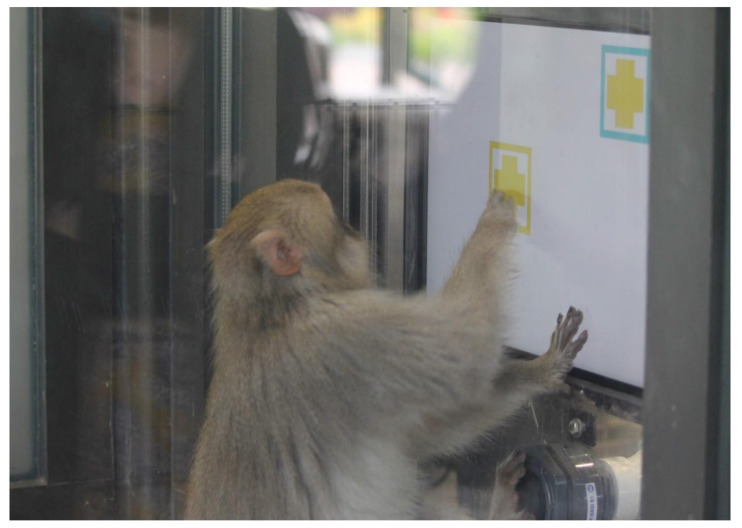
A photograph of one of the Japanese macaque subjects participating in a voluntary touchscreen test session at Lincoln Park Zoo. Here, the subject’s target stimulus-border color is yellow and she is selecting the correct stimulus in a congruent test trial in Test Phase 1.

Similar to the apes, we tested the Japanese macaques via two touchscreen testing booths that were connected directly to their outdoor exhibit and which were in view of zoo guests [16]. Each booth measured 216 cm × 114 cm × 122 cm and housed a touchscreen monitor (Figure 1) and the macaques could access either of the two adjacent booths via a swinging door. For each subject, we initiated a session when a macaque entered a booth. We paused that session if another macaque entered the same booth or if the subject left the booth. Two macaques could participate simultaneously in adjacent booths, and we resumed a session when a macaque re-entered a booth if they had not yet completed the maximum number of trials for that day (as for the apes, the maximum number of daily trials varied across test phases, described below). A session was terminated if a subject completed the maximum number of trials allowed for the day.

For this study, we administered two training phases and two test phases (described in detail below). While the methodological details varied across these phases, our general protocol was the same. In every trial, we showed a subject two stimuli on a white screen (all stimuli were 720 × 720 pixels). To help mitigate potential side biases in subjects’ selections, the stimuli appeared in random locations across the screen that differed across trials. There were 105 possible configurations in which the two stimuli could appear from a total of 15 locations on the screen (three rows by five columns). The two stimuli within each trial were always identical save for the color of the square border that framed them, such that one stimulus always had a yellow border (RGB 255, 215, 51) and one stimulus always had a blue border (RGB 17, 106, 200) (Figure 1). Regardless of the image shown within the border, the aim of this study was that subjects should only select one of the two stimuli, and that the “correct” response was for subjects to select the stimulus with their target stimulus-border color (Table 1, see Section 2.3. for details). For every trial in which a subject made a correct selection, by selecting the stimulus with their assigned target stimulus-border color, we reinforced them with a verbal bridge (“good job!”) and rewarded them with a preferred food reward (a grape for the chimpanzees and gorillas and a “Jungle Pellet” for the macaques [35,36]). We delivered the food rewards to the subjects via a plastic tube adjacent to the touchscreen. If the subjects selected the incorrect stimulus in a trial, we simply moved to the next trial (no reinforcement was given). Regardless of whether the subject selected the correct or incorrect stimuli, the inter-trial interval (ITI) remained the same (i.e., there was no “time out” for incorrect responses).

This study was reviewed and approved by the Lincoln Park Zoo Research Committee (approval number: 2017-004), which provides oversight for all animal research at the institution. This research adhered to all legal requirements in the United States of America and to the American Society of Primatologists’ Principles for the Ethical Treatment of Non-Human Primates. All testing was voluntary on the part of the primates and they were never deprived of food or water. Lincoln Park Zoo animal care, veterinary, and nutrition staff approved all foods prior to commencement of research, and all foods were a regular part of subjects’ diets.

### 2.3. Training Phase 1: Color Discrimination

**Figure 2:**
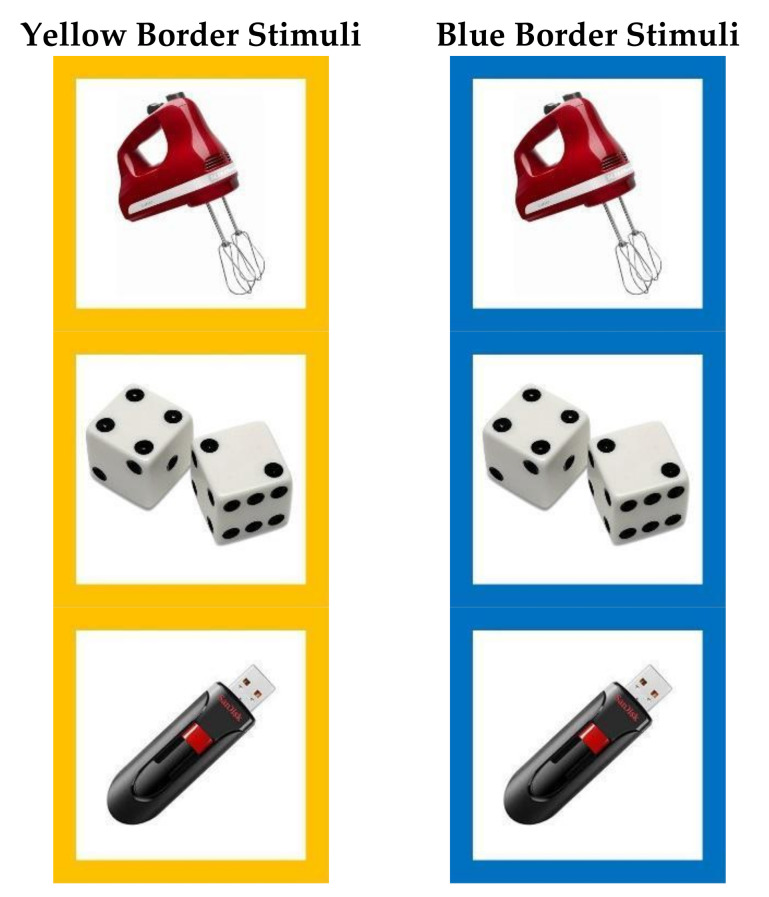
Example stimuli for Training Phase 1 and Phase 2. Each stimulus was a human-made object unfamiliar to the subjects presented on a white background. The stimuli were created as pairs of two identical photographs, one with a yellow border and one with a blue border.

The aim of the two training phases was to train the subjects on the association between selecting one of the two stimuli and getting a reward so as to condition them to select only their target stimulus-border color (i.e., to teach them the “correct” answer). Following Allritz et al. [27], for Training Phase 1, we created 50 stimuli that were full-color photographs of human-made objects, which we did not believe were familiar to the subjects, presented on a white background (Figure 2). We selected unfamiliar objects with the aim that all the stimuli would be neutral for all the subjects. Additionally, we were careful to select photographs that were devoid of yellow or blue. We then created two versions of each stimulus that were identical except for the color of a square border at the perimeter of each image: one stimulus had a yellow border and one had a blue border (Figure 2).

In each trial, we presented a matched pair of stimuli that only differed by the border color (yellow or blue). We pseudo-randomly assigned the subjects one of the border colors as their target stimulus-border color. Of the six chimpanzees, we attempted to train three subjects to select yellow and three to select blue; of the seven gorillas, we attempted to train four to select yellow and three to select blue; and, of the seven macaques, we attempted to train four to select yellow and three to select blue (Table 1). Each subject was tested in sessions of up to 50 trials, with each potential 50 stimuli pairing presented once within a session, although as testing was voluntary on the part of the subjects, we stopped some sessions early. We set the ITI for the apes to 4 s but to 8 s for the macaques to allow the researcher to more easily test two monkeys simultaneously in the adjacent testing booths.

We tested each subject in Training Phase 1 until they had been exposed to each of the 50 stimuli four times and continued testing until they reached criterion, which we set as them selecting the correct stimuli in ≥70% of trials in two consecutive sessions, each of ≥30 trials. Thus, the quickest way for a subject to reach criterion would be to complete 200 trials, in which they saw each stimulus four times and performed above chance in their selections for the two final sessions. We continued Training Phase 1 with each subject until they reached criterion, and moved on to Training Phase 2, or until they had completed 2500 trials, at which point we excluded them from further testing.

Testing for Training Phase 1 was conducted between June 2018 and October 2019. Of the 19 subjects, 10 (one chimpanzee, three gorillas, and six macaques) passed Training Phase 1 (Table 1). These 10 subjects were next tested in Training Phase 2. The remaining nine subjects each completed 2500 trials without reaching criterion and so were excluded from the remainder of this study.

### 2.4. Training Phase 2: Color Transfer Task

Following Allritz et al. [27], we tested all 10 subjects that passed Training Phase 1 color discrimination on a second training phase to confirm that they had learned to preferentially select one stimulus-border color over the other. The methods for this second training phase were identical to those described for Training Phase 1, except that we created 50 new pairs of stimuli with a novel set of 50 photographs of unfamiliar human-made items (Figure 2). We continued Training Phase 2 with each subject until they reached criterion and moved on to Test Phase 1 or until they had completed 2500 trials, at which point we excluded them from further testing. We conducted Training Phase 2 between June 2018 and July 2019. All of the 10 subjects that reached criterion in Training Phase 1 also reached criterion in Training Phase 2. Therefore, we next tested those 10 subjects in Test Phase 1.

### 2.5. Test Phase 1: Color-Interference Stroop Task

In Test Phase 1, as in the training phases, we presented the subjects with pairs of stimuli in each trial that only differed by their border color, and we only rewarded subjects for selecting stimuli that had their target stimulus-border color. However, unlike the training stimuli, the test stimuli were designed to elicit a Stroop effect.

**Figure 3:**
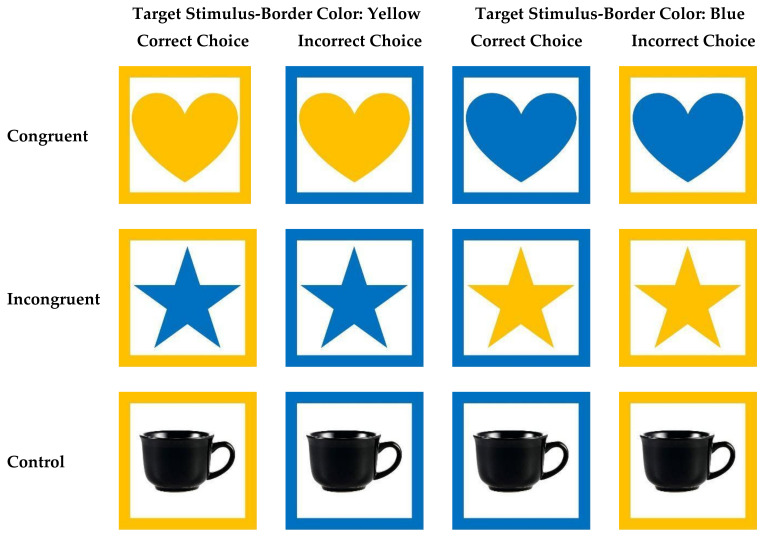
Examples of stimuli used in Test Phase 1 showing potential stimuli pairings for the three conditions (congruent, incongruent, and control). In congruent trials the color of the shape matched the subject’s target stimulus-border color for both target and distractor, while in incongruent trials the color of the shape is the opposite to that of the subject’s target stimulus-border color. In control trials, the images were neutral black and white photographs of human-made objects.

Following the methods of Allritz et al. [27], the test stimuli were four solid geometric shapes (a cross, a heart, a square, and a star) in the same shade of yellow and blue as the borders around each stimulus. None of the subjects had experience with these shapes as stimuli in previous touchscreen tasks and so all were novel to the subjects at the start of this study. This resulted in 16 unique stimuli with each shape-border color combination (e.g., for the star shape the combinations were: blue star with blue border, blue star with yellow border, yellow star with blue border, and yellow star with yellow border). The presentation of the pairs of test stimuli created two different test conditions: congruent trials and incongruent trials (Figure 3). In a congruent trial, the color of the shapes was the same as the subject’s target stimulus-border color, while in incongruent trials the color of the shapes was the opposite of the subject’s target stimulus-border color (Figure 1, Figure 3). Note, our methods differed to those used by Allritz et al. [27] in which the two images shown in each trial differed by both stimulus color and border color (see Figure 1b in [27] for examples of the stimuli combinations used in that study).

In addition to the test stimuli, we also created 10 control stimuli. These were a random selection of five of the photographs that we presented to the subjects in Training Phases 1 and 2 (specifically, they were photographs of a bicycle, a fidget spinner, a fork, a coffee cup, and a shovel) all presented on a white background and with yellow and blue borders (Figure 3). Following Allritz et al. [27], we converted these photographs to black and white images to make them more distinct from the test stimuli, to eliminate any potential interference from the color of the stimulus within the border, and to be less visually arousing than the test stimuli. As for test trials, in control trials one of the five black-and-white photographs was presented twice, once with a yellow border and once with a blue border, and the subject had to select the image with their target stimulus-border stimulus color.

In Test Phase 1, we tested each subject with 160 test trials (80 congruent trials and 80 incongruent trials) and 80 control trials, presented together in a randomized fashion across eight sessions, with a maximum of 30 trials per session.

We set the ITI for the apes at 4 s, except for one gorilla who was tested with a 3 s ITI for all his trials. As described for the training trials, at Lincoln Park Zoo we typically test the macaques with an 8 s ITI to allow the experimenter to more easily test two monkeys simultaneously in the adjacent testing booths. Accordingly, we initially tested two of the macaques in the group with an 8 s ITI. However, as Stroop tasks typically employ short ITIs, we wanted to explore the influence of ITI length on the monkeys’ responses. Therefore, we made the decision to try to test these two monkeys with a shorter ITI after they had completed their 240 Test Phase 1 trials to determine whether it was still feasible for the experimenter to administer the testing. We found it was. So, after piloting the shorter ITI with these two monkeys, we tested the rest of the macaques with 240 trials with the standard 8 s ITI and 240 trials with a 3 s ITI. To avoid order effects, we counterbalanced these ITI conditions across subjects such that half the monkeys first completed the 240 trials with the 8 s ITI and then completed the 240 trials with the 3 s ITI while the other monkeys were tested in the reverse order.

Testing for Test Phase 1 was conducted between August 2018 and October 2019. All four apes completed Test Phase 1, each completing 240 trials run over eight 30-trial sessions per subject. All six macaques completed the 240 trials of Test Phase 1 with an 8 s ITI, having completed an average of 22.86 trials per session (s.d. = 12.10), and five also completed all 240 trials with a 3 s ITI, but the final monkey (Ono) was tested with only 210 trials in this condition due to experimenter error (in this condition the macaques completed an average of 27.63 trials per session, s.d. = 9.11). After we completed Test Phase 1, we tested each subject in an unrelated study before proceeding to Test Phase 2. We did this to avoid ceiling effects in their selection of their target stimulus-border color in the two test phases (*sensu* [27]). The average delay between when subjects completed Test Phase 1 and started Test Phase 2 was 58 days (s.d. = 32.74).

### 2.6. Test Phase 2: Emotional Stroop Task

**Figure 4:**
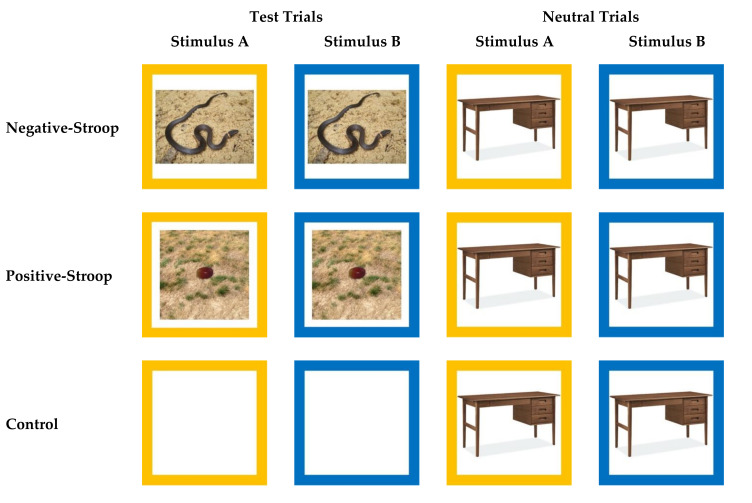
Stimuli categories across trial types and conditions in Test Phase 2. Within each trial, stimuli only differed by their border colors, but within each test condition (negative Stroop, positive Stroop, and Control) two trial types were presented to subjects (test trials and neutral trials) (Table 2). This figure provides examples of the stimuli presented across trial types within conditions, but note that different images were used across trials and conditions as described in the text. In this figure, images in the Stimulus A columns represent examples of correct choices for subjects whose target stimulus-border color was yellow, while images in the Stimulus B columns represent examples of correct options for subjects whose target stimulus-border color was blue.

In their study, Allritz et al. [27] used a pictorial “emotional Stroop” task with seven chimpanzees. They presented subjects with two sets of test stimuli and control stimuli. The test stimuli all showed photographs of humans, which were deemed either “non-negative” (photographs of familiar caretakers and of unfamiliar people) or “potentially negative” (photographs of a familiar veterinarian), while the control stimuli were white squares (see [27] for full methodological details). Similarly, we tested our subjects in two emotional Stroop conditions (negative and positive) and one control condition. In the negative-Stroop condition, we presented negative stimuli (photographs of snakes), in the positive-Stroop condition, we presented positive stimuli (photographs of a preferred food), and in the control condition, following Allritz et al. [27], we presented blank stimuli (i.e., a blue or yellow border around a white square) (Figure 4).

For the negative-Stroop condition stimuli, we chose to use photographs of snakes because snakes have been shown to be aversive stimuli for primates, as demonstrated by the snake detection effect [37,38] in which primates detect snakes more quickly than other stimuli in visual search tasks (e.g., [39,40]). Furthermore, photographs of snakes have previously been used in pictorial tests of emotional Stroop effects with humans [41]. We created 30 different negative stimuli, each of which was a unique full color photograph of a snake on a natural background that we gathered via a Google Image search (Figure 4). While all these stimuli were novel to the subjects at the start of testing, our previous research with these subjects had shown that they could spontaneously interpret photographs of real-life stimuli [35], and so we were confident in using these novel snake stimuli for Test Phase 2. As for the color-interference task, we created two versions of each stimulus, one with a yellow border and one with a blue border (Figure 4).

For the positive stimuli, we used a photograph of a grape for the apes and a photograph of a peanut for the macaques (we had taken these two photographs to use as stimuli in a previous test of the primates’ food preferences, which also revealed these foods to be highly preferred foods for the apes and macaques respectively [35,36]). To create positive stimuli that were as similar to the negative stimuli in terms of visual complexity, we superimposed the photograph of the food onto naturalistic backgrounds. We created 30 different versions of both the grape and peanut positive stimuli by using 30 distinct backgrounds (see Figure 4 for an example). These backgrounds varied in shade and color, like the backgrounds included in the photographs of the snakes that we used as the negative stimuli (i.e., we included equal numbers of grass, sand, and stone backgrounds for the food photographs as were shown in the snake photographs). Once we had created the 30 different positive stimuli for both apes and macaques, we made two versions of each stimulus, one with a blue border and one with a yellow border (Figure 4).

In every session, we showed subjects test trials (a pair of negative, positive, or control stimuli) and neutral trials (pairs of neutral stimuli) (Table 2). Following McKenna and Sharma [22], we blocked the trial presentation within a session such that we always showed the subjects three consecutive neutral trials followed by three consecutive test trials (snake, food, or control), repeated to create 30 total trials (15 neutral trials and 15 test trials) per session (see also [27]). For the neutral trial stimuli, we used images of 30 of the human-made object stimuli, randomly selected from the stimuli used in Training Phase 1 and 2 (Figure 4). Thus, in negative-Stroop condition sessions we presented snake and neutral trials, in positive-Stroop condition sessions we presented food and neutral trials, while in control condition sessions we presented control and neutral trials (Table 2). Within a session, we never showed the subject the same stimulus twice (although note, there was only one version of the control stimulus so this was used repeatedly) and subjects only experienced one test condition (negative Stroop, positive Stroop, or control) per session. In total, we tested each subject in eight sessions (240 trials) and the ITI was set at 3 s for all subjects.

The first session that all subjects completed in Test Phase 2 was a control condition session. If the subject met criterion in this session (selecting stimuli with their target stimulus-border color above chance), then on the next testing day we proceeded to either a negative-Stroop or positive-Stroop condition session. However, if they did not meet criterion in this first control session, we repeated the control condition the next day. Most (9/10) subjects met criterion in the first control condition session and the remaining subject met criterion in their second session. Following this first control session, we tested the subjects in either a positive-Stroop condition (food and neutral stimuli trials) or a negative-Stroop condition (snake and neutral stimuli trials), counterbalanced across subjects. We repeated this alternating presentation of control and emotional Stroop-condition sessions until each subject had completed eight sessions (four control, two negative-Stroop, and two positive-Stroop). Thus, while half the subjects started with the negative-Stroop condition (i.e., control-negative-control-positive-control-negative-control-positive), the other subjects were tested in the alternative sequence of sessions (i.e., control-positive-control-negative-control-positive-control-negative).

Testing for Test Phase 2 was conducted between January and November 2019. All ten subjects completed Test Phase 2. While we had intended for all subjects to complete four control sessions and four Stroop sessions (two positive-Stroop, two negative-Stroop), due to experimental error, two macaques (Ono and Otaru) only received three control sessions. The apes completed all 240 trials over the course of eight 30-trial sessions while the macaques completed an average of 26.60 trials per session (s.d. = 6.97). As a result of the voluntary nature of our testing protocol, two macaques (Nagoya and Otaru) did not complete all 30 trials in their first presentation of the positive-Stroop test session and so were provided with additional trials at the end of this study to ensure they each received the total 60 positive-Stroop trials.

### 2.7. Data Preparation and Analyses

To analyze the subjects’ relative accuracy across conditions in the color-interference Stroop task (Test Phase 1) and emotional Stroop task (Test Phase 2), we used binomial generalized linear mixed models (GLMMs) using the lme4 package [42] in R version 4.0.2 [43]. To do so, first we coded each trial as correct (1), in which the subject selected their target stimulus-border color irrespective of the image within the border, or incorrect (0), in which they selected the alternative stimulus. Next, we excluded subjects’ trials in which they responded with a latency 2.5 SD above their average response time (*sensu* [16]). In doing so, for Test Phase 1 we excluded 144/3809 trials (3.78%) and we excluded 72/2, 370 trials (3.04%) for Test Phase 2.

For Test Phase 1, we explored the subjects’ relative selection of correct stimuli in the congruent, incongruent, and control trials. In our GLMM we included subject ID as a random effect and trial type (congruent, incongruent, and control) and species (ape and monkey) as fixed effects (we combined the apes into a single category as we only had data from one chimpanzee). For the macaques only, we also ran an additional analysis to explore the effect of ITI length (see Methods 2.5). To do so, we ran a binomial GLMM but we included trial type (congruent, incongruent, and control) and ITI length (3 s and 8 s) as fixed effects and we included subject ID as a random effect.

For Test Phase 2, we analyzed the subjects’ accuracy in three ways via binomial GLMMs. First, we compared the subjects’ accuracy across test trial type (i.e., comparing their accuracy in test trials in each of the three conditions, excluding their responses in neutral trials) (see Table 2). We included “trial type” (snake, food, and control) and species (ape and monkey) as fixed effects and subject ID as a random effect. Second, and running a model for each of the three conditions (negative Stroop, positive Stroop, and control), we compared the subjects’ relative accuracy in test trials (snake, food, or control) to their accuracy in neutral trials within the same condition. We included trial type and species as fixed effects and subject ID as a random effect. Third, to assess whether the subjects’ accuracy in neutral trials was influenced by the emotionally valent test trials they were interspersed with in the same test session, we compared the subjects’ rates of accuracy in neutral trials across the three conditions (negative Stroop, positive Stroop, and control). We included “test condition” and species as fixed effects and subject ID as a random effect.

In addition to analyzing the accuracy of the primates’ responses across test trials and conditions in Test Phase 1 and Test Phase 2, we also examined their response latencies. As the primates’ response times were not normally distributed (they were positively skewed), we log(10) transformed the response times (*sensu* [16]) and used the log(10) transformed data for further analysis. To compare the subjects’ response latencies to select correct stimuli in the three conditions in Test Phase 1, and by trial type and condition in Test Phase 2, we used a linear model using the lmerTest package [44] in R [43] following the model structure described for the GLMMs.

We created all data visualizations using ggplot2 [45] in R version 4.0.2 [43].

## 3. Results

### 3.1. Training Phase 1: Color Discrimination

Of the 19 subjects, 10 (one chimpanzee, three gorillas, and six macaques) passed Training Phase 1 (Table 1). An equal number of these subjects were trained with the blue stimulus-border color (1 chimpanzee, 1 gorilla, 3 macaques) as with yellow (2 gorillas, 3 macaques), suggesting that the primates did not more easily learn one color over the other. The fewest number of trials in which the subjects could reach criterion was 200. The chimpanzee required 2259 trials to reach criterion, having completed an average of 47.06 trials per session (s.d. = 11.04). The three gorillas required an average of 1853.00 trials (s.d. = 912.43) to reach criterion, having completed an average of 49.63 trials per session (s.d. = 6.77). The six macaques required an average of 726.83 trials (s.d. = 647.74) to reach criterion, having completed an average of 37.27 trials per session (s.d. = 16.84).

### 3.2. Training Phase 2: Color Transfer Task

All 10 subjects that reached criterion in Training Phase 1 also reached criterion in Training Phase 2, demonstrating that they had reliably learned to preferentially select their target stimulus-border color. In Training Phase 2, the chimpanzee required 200 trials to reach criterion—the fewest number of trials in which a subject could reach criterion—having completed four sessions of 50 trials. One gorilla also reached criterion in only 200 trials but on average the three gorillas required 766.67 trials (s.d. = 775.13) to reach criterion, having completed an average of 50.00 trials per session (s.d. = 0.21). The six macaques required an average of 361.17 trials (s.d. = 130.42) to reach criterion, having completed an average of 38.91 trials per session (s.d. = 14.98).

### 3.3. Test Phase 1: Color-Interference Stroop Task

**Figure 5:**
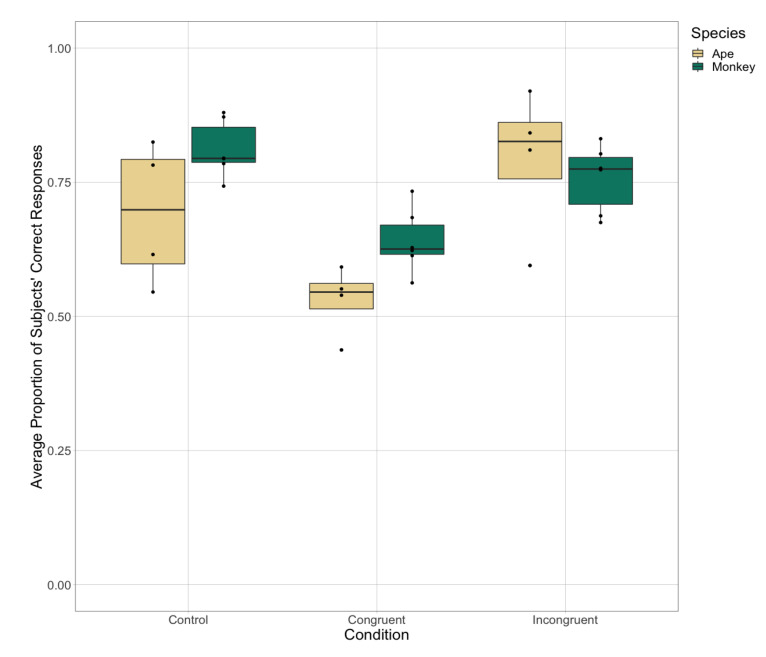
The subjects’ average accuracy in selecting the stimuli with their target stimulus-border color across the three experimental trial types in Test Phase 1 when the ITI was short (3 s or 4 s).

In Test Phase 1, when considering only trials in which the ITI was short (3 s or 4 s), there was a significant effect of condition and species on the subjects’ accuracy in selecting their target stimulus-border color, as well as interaction effects between species and condition (Table 3, Figure 5). Given the main effect of species, we analyzed the apes’ and monkeys’ responses separately to explore their accuracy across conditions. The apes and monkeys both performed worst in the congruent condition. The apes made significantly more accurate responses in the incongruent condition as compared to in the congruent (Z = 6.80, *p* < 0.001 [CI 0.89, 1.61]) and control (Z = 2.82, *p* = 0.004 [CI 0.16, 0.90]) conditions. They also made significantly more accurate responses in the control condition as compared to in the congruent condition (Z = −4.19, *p* < 0.001 [CI −1.05, −0.38]). The monkeys made significantly more accurate responses in the control condition as compared to the incongruent condition (Z = −2.08, *p* = 0.038 [CI −0.66, −0.02]) and congruent condition (Z = −5.57, *p* < 0.001 [CI −1.19, −0.57]). They also made significantly more accurate responses in the incongruent condition as compared to the congruent condition (Z = 3.60, *p* < 0.001 [CI 0.24, 0.83]). While we found differences in subjects’ accuracy across conditions, there was no significant effect of species or condition (and no interaction effects) on the subjects’ response latencies across all trials in Test Phase 1 nor when only considering trials in which subjects selected the correct stimulus (i.e., their target stimulus-border color).

**Figure 6:**
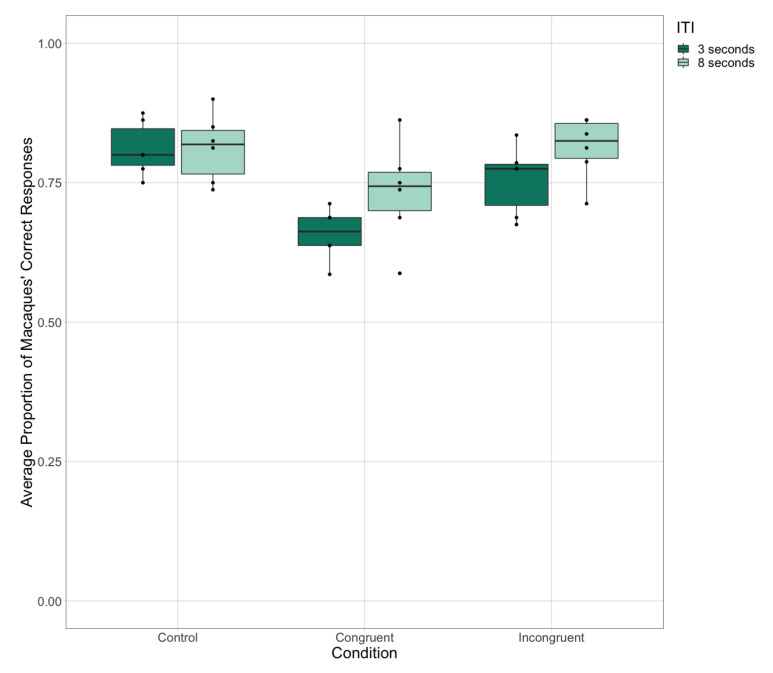
The macaques’ average accuracy across the three experimental trial types in Test Phase 1 as tested with short (3 s) and long (8 s) ITIs.

As described in the methods, we only tested the apes with a short ITI but we tested the macaques with both a short ITI (3 s) and a long ITI (8 s) across all conditions in Test Phase 1. Considering just the monkeys’ responses, we compared their accuracy across trial types and by ITI length. The model revealed a main effect of both trial type, reflecting the pattern of results described above, and ITI length, such that the monkeys were significantly more accurate in trials with a long, than with a short, ITI (Z = −3.22, *p* = 0.001 [CI −0.47, −0.12]) (Figure 6). This reflects the impact of ITI length on the subjects’ cognitive processing and their response to these stimuli.

### 3.4. Test Phase 2: Emotional Stroop Task

**Figure 7:**
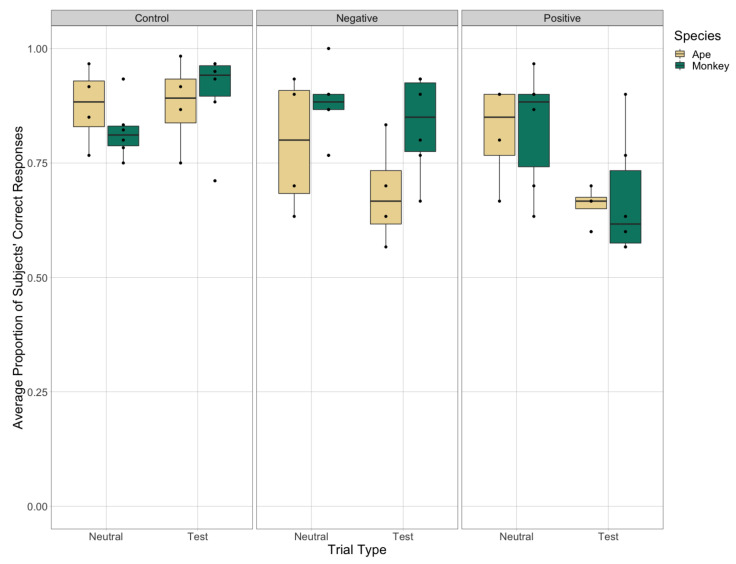
The subject’s average accuracy in neutral and test trials across the three experimental conditions (control, negative Stroop, and positive Stroop) in Test Phase 2. Here, test trials refer to the control, snake, and food trials in each of the three experimental conditions respectively (see Figure 4 and Table 2).

Considering only responses on test trials (Table 2), the primates made significantly fewer accurate responses in the negative (snake) and positive (food) Stroop trials as compared to in control trials (Table 4, Figure 7). There was no difference, however, in their accuracy across the two Stroop test trial types (snake and food), although there was an interaction effect such that the monkeys were more accurate in negative test trials (snake) than in positive test trials (food), whereas the opposite pattern was true for the apes. There was no significant effect of species or trial type (and no interaction effects) on the subjects’ response latencies across test trials in Test Phase 2, whether analyzing all test trials or only test trials in which subjects selected the correct stimulus with their target stimulus-border color.

Next, we compared the subjects’ accuracy across test and neutral trials within each of the three conditions (negative Stroop, positive Stroop, and control). In the control condition, there was no difference in the subjects’ accuracy between the neutral and control trials (Table 5, Figure 7). In contrast, in the positive-Stroop condition, both apes and monkeys were significantly more accurate in neutral trials than in test trials (food). Finally, in the negative-Stroop condition, the apes were significantly less accurate in test trials (snake) than neutral trials, whereas the monkeys showed high accuracy for both trial types in this condition (Figure 7). There was no significant effect of species or trial type (and no interaction effects) on the subjects’ response latencies in any of the three conditions in Test Phase 2, whether analyzing all trials or only when considering trials in which subjects selected the correct stimulus (i.e., their target stimulus-border color).

Finally, we explored if the subjects’ accuracy in neutral trials was impacted by the test trial types (snake, food, and control) also presented in each session (i.e., by condition) (Table 2). To do so, we compared the subjects’ accuracy in neutral trials across the three conditions (negative Stroop, positive Stroop, and control). While there was no effect of condition or species on the subjects’ accuracy in neutral trials, there was an interaction effect between species and condition, such that the apes were more accurate in the control condition than the monkeys, while the monkeys were more accurate in the negative-Stroop condition than the apes (Table 6, Figure 7). There was no significant effect of species or condition (and no interaction effects) on the subjects’ response latencies in neutral trials across conditions in Test Phase 2, whether analyzing all trials or only when considering neutral trials in which subjects selected the correct stimulus (i.e., their target stimulus-border color).

## 4. Discussion

In this study, we adapted a previously published pictorial Stroop paradigm [27] for use with three primate species. Of the subjects that passed pre-test training, the primates’ responses in the color-interference Stroop task suggested that they did not show a Stroop-like effect in response to incongruent stimuli (Test Phase 1), but we did find that the subjects’ performance was impacted by the inter-trial interval length. Specifically, and as reported previously for humans [46], a shorter inter-trial interval (3 s) was associated with macaques making more errors than in trials separated by longer intervals (8 s). Furthermore, we found that the primates did show a Stroop effect in response to both positively and negatively valenced stimuli in the emotional Stroop task (Test Phase 2), although there were differences between the apes and the monkeys in their response to the stimuli types. In addition to offering insights into primate cognitive processing and affect, these results provide further validation to the original study [27], which was conducted with chimpanzees housed at a different zoo, and also shows its applicability for use with other primate species. We did find, however, some intriguing species differences in subjects’ responses to the stimuli, as well as differences in how our subjects responded as compared to the chimpanzees tested by Allritz et al. [27], which warrant further discussion.

In our color-interference Stroop task (Test Phase 1), we found that the primates showed reduced accuracy in the congruent condition (in which the stimulus color matched the subject’s target stimulus-border color). This is counter to our predictions and differs from previous work using similar tasks with primates (e.g., [27,29,30]). Specifically, we had predicted that, if the primates were to demonstrate a Stroop-like effect, they would show increased response times and/or reduced accuracy in incongruent trials but that their accuracy rates and response latencies would be comparable across control and congruent trials. We did not find evidence for interference in attention due to incongruent stimuli: both the apes and monkeys were more accurate in the incongruent condition than the congruent condition. While the primates’ high rates of accuracy in the incongruent trials may at first seem counterintuitive, we propose that this is a result of our particular methods in which it may have been easier for subjects to identify the target stimulus in incongruent condition trials in our version of the Stroop task. That is, unlike in the study by Allritz et al. [27], in the incongruent trials in our study, only one of the two stimuli on the screen contained the subject’s trained target color (Figure 3, compare with Figure 1b in [27]). The upshot of this may have been that it was easier for the subjects to discriminate between the two stimuli in the incongruent trials and identify the correct stimulus than it was in congruent trials. Similarly, in the control trials, in which the stimuli were always black and white, it also would be easier to identify the target stimulus. In congruent trials, however, both stimuli contained the target color (Figure 3), potentially making it more difficult for the subjects to differentiate the stimuli and reliably select the correct one. While we believe that this explanation likely explains the primates’ reduced success in congruent trials, it is notable that the primates did not show increased response latencies in congruent trials, as compared to the other trial types in which stimuli discrimination was potentially easier.

In the color-interference Stroop task, we found no differences in the primates’ response latencies across trial types. The nonsignificant difference in subjects’ response latencies across conditions in Test Phase 1 (and also in Test Phase 2) could be due to a number of factors. For example, we tested subjects at a zoo where many other potential distractions are present in the environment [17]. Additionally, it may be that we ran too few trials to detect differences in responses latencies across conditions, or perhaps the inclusion of a high proportion of congruent trials may have biased the subjects’ responses overall [47]. However, these two potential explanations do not explain the differences in our subjects’ responses with those tested by Allritz et al. [27], who followed comparable protocols. One key difference between our subjects and the chimpanzees tested by Allritz et al. [27] is that our subjects had previously completed numerous touchscreen tasks [16,35,36,48,49,50], whereas the chimpanzees tested by Allritz et al. [27] were naïve to touchscreens at the start of their study. The familiarity of our subjects with touchscreen testing may have led to ceiling effects in their response latencies, masking any (potential) differences across conditions. Furthermore, previous work with chimpanzees has also reported Stroop-like effects expressed in terms of reduced accuracy but not via differential response latencies [28], highlighting the fragile nature of this effect.

In Test Phase 2, which created interference via positively and negatively valenced stimuli (i.e., an emotional Stroop task), we found evidence for a Stroop-like effect. Specifically, in the positive-Stroop condition, both the apes and monkeys showed significantly lower accuracy in trials that presented positively valenced stimuli (a preferred food) as compared to neutrally valenced trials. In the negative-Stroop condition, however, only the apes showed a Stroop-like effect, responding less accurately to test trials that showed a snake as compared to neutral trials. At this time, we do not have a clear explanation for what might be driving this pattern in our results (i.e., why only the positive condition induced a Stroop-like effect for both apes and monkeys or why only apes showed a Stroop-like effect in response to negative stimuli), although other researchers have reported differences across primate species in their response to snake stimuli [51]. However, we do wish to consider the specific test stimuli we used. Not only were these test stimuli of different valences, but they were also of different levels of familiarity to the subjects we tested. Specifically, the subjects had never seen any of the snake photographs before the start of testing but the food photographs were familiar to the subjects as they had been presented as part of previous tests of these primates’ food preferences [35,36], although not in combination with the backgrounds we added to the stimuli here. Therefore, these food stimuli may have been more potent and the subjects’ prior experience with these stimuli may have influenced their selection of them. Additionally, while the primates performed worse in the emotional Stroop trials as compared to neutral trials or control trials, as with the color-interference Stroop task, we found no difference in the primates’ response latencies across conditions in this emotional Stroop task. This is in contrast to previous work with nonhuman primates that has shown increased response times to emotionally valenced stimuli [15,16,27], although such results are mixed across settings and species (e.g., [16,17]).

One key limitation of our study is that only 10 of the 19 subjects passed the training required before we could test them in the two Stroop tests. This high attrition rate meant that we could only assess half our potential subjects. Although Allritz et al. [27] tested more apes than we were able to, they also had to exclude potential subjects from their study. Of 10 chimpanzees, only six passed the initial training phase in the study by Allritz et al. [27]. The remaining four chimpanzees did not reach criterion even after having completed 4000 trials (note, we set our exclusion criterion to 2500 trials maximum), but two of those four chimpanzees ultimately reached criterion after completing additional training. Perhaps if we had extended our exclusion criterion beyond 2500 trials, or followed a less stringent pass criterion (e.g., >70% success in just one, rather than two, training sessions), we may have been able to test more subjects in later phases of this study. Regardless, this highlights the extensive pre-test training required, which is time consuming and leads to potential subjects being excluded from testing. Therefore, and as noted by Bethell et al. [26], this limits the practicality of this method for testing primates’ responses to emotional stimuli as extensive training is required, data are time consuming to gather, and responses cannot be reliably obtained for all animals. We therefore encourage researchers to explore other ways to test emotional influences on cognitive processing, especially if the aim is to gain insights into animal welfare, for which faster or more reliable metrics might be preferred.

Beyond practical concerns with our approach, we also want to acknowledge that despite interest in exploring and defining animals’ emotional experiences [11,52,53], there is no clear correspondence between animals’ internal experiences and measurable outputs (behavioral or physiological changes) [54]. Moreover, Bliss-Moreau and Rudebeck [55] note that animals’ internal experiences can be defined in terms of both arousal and valence, but that *moods* require a conscious experience. Therefore, for cross-species considerations, they propose that *temporally extended affective states* offer a more useful operational term for describing the internal affective states that animals experience. Thus, while others have explored different approaches to test attentional biases in captive primates for applied reasons (e.g., [15,16,17,18,56,57]), this is still an area of research that warrants further attention both in terms of the methods and terminology used [9].

## 5. Conclusions

While all subjects readily participated in the voluntary test sessions, only half (10/19) passed the pre-test training phase. Moreover, while all of the six macaques passed the training, only four of the 13 apes did so. At this time, we do not have a clear explanation for this difference or whether it reflects species differences or variations related to our testing protocols. Of those primates that passed the pre-test training, we found that both apes and monkeys showed a Stroop-like response to positively valenced stimuli, but that only apes also showed such a response to negatively valenced stimuli. The approach we used to test the potential interplay between cognitive processing and emotional content was developed from a foundation of research with human participants. Furthermore, the use of touchscreens allowed us to deploy the same protocol with three species allowing for meaningful cross-species comparisons. Although we did not do so here, it is likely that this protocol could be further adapted to allow for the presentation of different kinds of stimuli to facilitate more detailed explorations of primates’, and potentially other species’, decision making biases. In spite of this, due to the long training and testing period, combined with our inability to test all potential subjects, we do not believe that this protocol is nimble enough for regular welfare assessments. However, while this protocol may not have allowed us to *assess* welfare, it may well have enabled us to enhance the subjects’ welfare. There is increasing evidence that cognitive testing can be enriching (e.g., [58,59]), especially under the voluntary testing protocols (e.g., [34,60]), as we used here. In this way, future tests of primate welfare could be developed that not only more reliably and efficiently allow for evaluations of primates’ temporally extended affective states, but also enhance the well-being of the subjects being tested [61,62].

## Figures and Tables

**Table 1:** The 19 subjects and which of the two training phases each subject passed and which of the two test phases they completed. This table also shows which stimulus-border color each subject was assigned as their target.

Species	Subject (Sex, Age)	Social Group	Border Color	Training Phase 1	Training Phase 2	Test Phase 1	Test Phase 2
*Pan* *troglodytes*	Cashew (F, 33)	1	Yellow	Failed			
	Chuckie (F, 18)	1	Yellow	Failed			
	Hank (M, 27)	1	Blue	Failed			
	Kathy (F, 27)	1	Blue	Passed	Passed	Completed	Completed
	Magadi (F, 27)	2	Yellow	Failed			
	Optimus (M, 19)	1	Blue	Failed			
*Gorilla* *gorilla* *gorilla*	Amare (M, 12)	3	Blue	Failed			
	Azizi (M, 14)	3	Yellow	Passed	Passed	Completed	Completed
	Kwan (M, 29)	4	Blue	Passed	Passed	Completed	Completed
	Mosi (M, 11)	3	Blue	Failed			
	Patty (F, 5)	4	Yellow	Failed			
	Rollie (F, 21)	4	Yellow	Failed			
	Umande (M, 12)	3	Yellow	Passed	Passed	Completed	Completed
*Macaca* *fuscata*	Izumi (F, 13)	5	Yellow	Passed	Passed	Completed	Completed
	Mito (F, 12)	5	Blue	Passed	Passed	Completed	Completed
	Miyagi (F, 13)	5	Yellow	Passed	Passed	Completed	Completed
	Nagoya (F, 2)	5	Blue	Passed	Passed	Completed	Completed
	Ono (F, 13)	5	Yellow	Passed	Passed	Completed	Completed
	Otaru (F, 2)	5	Blue	Passed	Passed	Completed	Completed

**Table 2:** Subjects were tested in three conditions in Test Phase 2. In each condition, subjects received equal numbers of test trials and neutral trials, presented in blocks of three trials. Examples of the stimuli for the different test and neutral trials are shown in Figure 4.

Condition	Trial Type	
	Test	Neutral
Negative-Stroop	Negative (snake)	Neutral (human-made object)
Positive-Stroop	Positive (food)	Neutral (human-made object)
Control	Control (white square)	Neutral (human-made object)

**Table 3:** The results of the general linear mixed model comparing the relative influence of species (ape and monkey) and trial type (control, congruent, and incongruent), and any interactions between them, on the subjects’ accuracy in Test Phase 1. Subject ID was included as a random effect.

Comparison	SD	Z	*p*-Value	CI (2.5%)	CI (97.5%)
Control vs. Congruent	0.17	−4.19	<0.001	−1.04	−0.38
Control vs. Incongruent	0.19	2.81	0.005	0.16	0.90
Congruent vs. Incongruent	0.18	6.76	<0.001	0.88	1.59
Species	0.24	2.64	0.008	0.17	1.11
Control × Congruent × Species	0.23	−0.75	0.452	−0.63	0.28
Control × Incongruent × Species	0.25	−3.49	<0.001	−1.36	−0.38
Congruent × Incongruent × Species	0.24	−2.94	0.003	−1.16	−0.23

**Table 4:** The results of the general linear mixed model comparing the relative influence of species (ape and monkey) and experimental trial type (negative snake photographs, positive food photographs, or blank control stimuli), and any interactions between them, on the subjects’ accuracy in Test Phase 2. Subject ID was included as a random effect.

Comparison	SD	Z	*p*-Value	CI (2.5%)	CI (97.5%)
Control vs. Positive	0.28	−4.37	<0.001	−1.79	−0.68
Control vs. Negative	0.28	−4.29	<0.001	−1.76	−0.66
Positive vs. Negative	0.28	0.09	0.929	−0.53	0.58
Species	0.39	1.11	0.267	−0.33	1.20
Control × Positive ×Species	0.38	−1.16	0.248	−1.19	0.31
Control ×Negative × Species	0.40	1.18	0.238	−0.31	1.27
Positive × Negative × Species	0.39	2.37	0.018	0.16	1.68

**Table 5:** The results of the general linear mixed model comparing the relative influence of species (ape and monkey) and trial type (test and neutral), and any interactions between them, on the subjects’ accuracy in each of the three test conditions in Test Phase 2. Subject ID was included as a random effect.

Condition	Comparison	SD	Z	*p*-Value	CI (2.5%)	CI (97.5%)
Control	Trial Type ^1^	0.24	−1.34	0.180	−0.78	0.15
	Species	0.44	0.87	0.386	−0.48	1.24
	Trial ^1^ × Species	0.33	−1.35	0.178	−1.08	0.20
Negative Stroop	Trial Type ^1^	0.24	3.83	<0.001	0.44	1.37
	Species	0.30	2.19	0.028	0.09	1.68
	Trial ^1^ × Species	0.33	−2.78	0.005	−1.57	−0.27
Positive Stroop	Trial Type ^1^	0.24	−3.91	<0.001	−1.39	−0.46
	Species	0.32	−0.04	0.968	−0.64	0.61
	Trial ^1^ × Species	0.31	0.02	0.982	−0.59	0.61

^1^ The two trial types in the control condition were control and neutral trials, the two trial types in the negative-Stroop condition were snake and neutral trials, while the two trial types in the positive-Stroop condition were food and neutral trials (Figure 4).

**Table 6:** The results of the general linear mixed model comparing the subjects’ accuracy in neutral stimuli trials in Test Phase 2 by condition (negative Stroop, positive Stroop, and control) and species (ape and monkey), and any interactions between them. Subject ID was included as a random effect.

Comparison	SD	Z	*p*-Value	CI (2.5%)	CI (97.5%)
Control vs. Positive	0.31	−1.47	0.143	−1.01	0.15
Control vs. Negative	0.31	−1.94	0.053	−1.19	0.01
Positive vs. Negative	0.33	−0.41	0.683	−0.79	0.52
Species	0.42	0.09	0.925	−0.78	0.86
Control × Positive × Species	0.40	−1.30	0.193	−1.29	0.26
Control × Negative × Species	0.41	2.87	0.004	0.38	2.00
Positive × Negative × Species	0.46	1.48	0.140	−0.22	1.56

## Data Availability

Data are provided in Material S1.

## Supplementary Materials

The following are available online at https://www.mdpi.com/2076-2615/11/3/588/s1, Material S1: The raw data for Test Phase 1 (color-interference Stroop test) and Test Phase 2 (emotional Stroop test) for all 10 subjects that passed the pre-test training.
